# Effect of Multiplicity Fluctuation in Cobalt Ions on Crystal Structure, Magnetic and Electrical Properties of NdCoO_3_ and SmCoO_3_

**DOI:** 10.3390/molecules25061301

**Published:** 2020-03-12

**Authors:** Vyacheslav A. Dudnikov, Yuri S. Orlov, Leonid A. Solovyov, Sergey N. Vereshchagin, Sergey Yu. Gavrilkin, Alexey Yu. Tsvetkov, Dmitriy A. Velikanov, Michael V. Gorev, Sergey V. Novikov, Sergey G. Ovchinnikov

**Affiliations:** 1 Kirensky Institute of Physics, Federal Research Center KSC SB RAS, 660036 Krasnoyarsk, Russia; slad63@yandex.ru (V.A.D.); velikanov@iph.krasn.ru (D.A.V.); sgo@iph.krasn.ru (S.G.O.); 2 Institute of Engineering Physics and Radio Electronics, Siberian Federal University, 660041 Krasnoyarsk, Russia; 3 Institute of Chemistry and Chemical Technology, Federal Research Center KSC SB RAS, 660036 Krasnoyarsk, Russia; leosol@icct.ru (L.A.S.); snv@icct.ru (S.N.V.); 4 Lebedev Physical Institute of the Russian Academy of Sciences, 119991, Moscow, Russia; gavrs@lebedev.ru (S.Y.G.); tsvetkov@lebedev.ru (A.Y.T.); 5 Ioffe Institute of the Russian Academy of Sciences, 194021 St. Petersburg, Russia; snpochta@gmail.com

**Keywords:** rare-earth cobalt oxides, multiplicity fluctuations, structural, magnetic, electrical, and dilatation properties

## Abstract

The structural, magnetic, electrical, and dilatation properties of the rare-earth NdCoO_3_ and SmCoO_3_ cobaltites were investigated. Their comparative analysis was carried out and the effect of multiplicity fluctuations on physical properties of the studied cobaltites was considered. Correlations between the spin state change of cobalt ions and the temperature dependence anomalies of the lattice parameters, magnetic susceptibility, volume thermal expansion coefficient, and electrical resistance have been revealed. A comparison of the results with well-studied GdCoO_3_ allows one to single out both the general tendencies inherent in all rare-earth cobaltites taking into account the lanthanide contraction and peculiar properties of the samples containing Nd and Sm.

## 1. Introduction

The unusual temperature dependence of the magnetic susceptibility and transport properties of LaCoO_3_ cobalt oxide [1,2,3,4] have led to the active study of RCoO_3_ cobaltites (R is a rare-earth element, RE) and their derivatives for half a century. A characteristic property of such compounds is their proximity to the spin crossover or the approximate equality of Hund’s energy $J_{H}$ and the 10Dq crystal field in the CoO_6_ octahedral complexes formed in a rhombohedral or rhombical distorted perovskite-like structure [5,6,7]. This leads to multiplicity fluctuations or thermal fluctuations of the spin value (we want to emphasize that the spin value fluctuations considered here should not be understood as spin fluctuations. The latter usually means fluctuations of the spin projection. Many years ago, Vonsovskii introduced a term «multiplicity fluctuations» to discuss a variation of the magnitude of the spin in the *d* shell [8]) and competition between the low-spin (LS, S = 0, t_2g_^6^) and high-spin (HS, S = 2, t_2g_^4^e_g_^2^) states of the Co^3+^ ion, with their intensity depending on the RE ionic radius (“lanthanide contraction”) and environmental factors such as temperature or pressure. It is this competition, combined with the original properties of the rare-earths by themselves, that causes the unique physical properties of the rare-earth cobalt oxides.

Usually the spin crossover is related to the different multiplets level crossing, under high pressure in many iron oxides it is rather abrupt at some critical pressure $P_{C}$ with the width of crossover dependent on temperature, for the Fe_x_Mg_1-x_O measurements at 5K found the width close to zero [9]. Near $P_{C}$ the spin gap defined as the HS and LS energy difference may be comparable to the thermal energy $k_{B}T$ and the spin crossover can be revealed in the temperature dependences of various material properties. It is the case of LaCoO_3_ and other cobaltites. Rare-earth cobaltites in contrast to iron oxides turn out to be in the LS- state already at T = 0 and at zero applied pressure; i.e., the spin crossover already occurs in the course of the formation of their structure owing to the «chemical pressure» determining the equilibrium volume of the unit cell.

The crystal field 10Dq, in contrast to the intraionic exchange interaction $J_{H}$, can vary depending on the interatomic metal-ligand distance. Hydrostatic or chemical pressure and stretching make it possible to influence the strength of the crystal field and, thus, the population of e_g_ and t_2g_ orbitals. In [10] authors reported an unambiguous demonstration of dimensionality control of *d*-orbital occupation with different symmetries (t_2g_ and e_g_) in atomically thin Mott insulator-band insulator oxide superlattices. The heating induced lattice expansion also vary the crystal field value and related smooth spin crossover effects are the subject of our paper.

The most studied cobaltite compound is LaCoO_3_ [11,12]. The ground state of cobalt ions is defined as the nonmagnetic LS- state without any doubt. A series of EPR experiments [13] and X-ray spectroscopy data [14] on LaCoO_3_ compositions indicate the cobalt ions transition from the low-spin state to the high-spin state with increasing temperature. This confirms the scheme of multi-electron level given in [15], where the ground LS- state is separated by the spin gap $\Delta_{S}$ from the HS- state splitted into sublevels with the total effective momentum $\tilde{J}$ = 1, 2, 3 due to the spin – orbit interaction. This scheme is consistent with the Tanabe - Sugano diagrams for transition metal ions in octahedral complexes developed in perovskite-like materials [16]. The approximate equality of the Curie constant at room temperature to the value given by S = 1 mimics the presence of the intermediate-spin (IS, S=1, t_2g_^5^e_g_^1^) states, but it follows from the smooth temperature dependence of the average magnetic moment of the mixed LS- and HS- states [17]. Multielectron calculations of the LDA+GTB electronic structure and the properties of LaCoO_3_ [17] using the term scheme [15] have shown that, due to multiplicity fluctuations, the effective spin is determined by a combination of LS- and HS- states and depends on temperature, therefore it is close to 1 near room temperature and the saturation S = 2 has been reached only at high temperatures up to 1000K.

The properties of other rare-earth cobaltites have been studiedless. However, the similar features both in magnetic susceptibility behavior χ(T), thermal dilatation α(T), heat capacity $C_{p}\left(T\right)$, electrical resistance ρ(T), have been observed for some RCoO_3_ compounds similar to the known for LaCoO_3_ [18,19,20,21,22,23,24,25]. With increasing the RE atomic number, the unit cell volume decreases, the additional chemical pressure increases, and the spin gap Δ_S_ increases, stabilizing the LS- state of cobalt ions to higher temperatures [26]. Moreover, the anomalies in the behavior of χ(T), α(T), $C_{p}\left(T\right)$, and ρ(T) shift to the region of higher temperatures and are smoother, giving a correlation between electromagnetic and thermodynamic properties. It should be noted that in most cases the magnetic transition temperatures of the ReCoO_3_ compounds associated with the magnetic moment ordering of *4f*- ions are low, $T_{N}$~ 1 K [27]. In the paramagnetic region, large RE moments make the main contribution to the magnetic susceptibility; however, a small Co^3+^ ions contribution can be distinguished only at high temperatures. This is probably the reason that most of the experimental work is devoted to the study of the thermodynamic and transport properties of cobaltites [5,7,28,29]. LaCoO_3_ is an exception, when total magnetic susceptibility is due to Co^3+^ ions. The magnetic properties of other rare-earth cobaltites have less been studied [30,31,32,33].

Considering the potential for extensive use of perovskite-like complex cobalt oxides as solid state oxide power sources [34,35,36], oxygen membranes [37], gas sensors [38], etc., a more detailed study of the rare-earth cobaltite properties seems to be appropriate.

In current paper structural, magnetic, electrical, and dilatation properties of the rare-earth cobaltites NdCoO_3_ and SmCoO_3_ have been studied and their comparative analysis was carried out. The influence of the multiplicity fluctuations of Co^3+^ ions on physical properties of the studied cobaltites is considered within a virtual crystal model. In this model previously proposed in the study of GdCoO_3_the average crystal volume at temperature T is determined by the volume superposition in LS- и HS- states with statistical weights given by the population of LS- and HS- terms [39]. Then, the maximum of anomalous thermal dilatation for GdCoO_3_was found at the temperature of about 750 K in [5]. A lower lanthanide compression for Nd^3+^ and Sm^3+^ ions as compared to Gd^3+^can be expected to enhance the effects of multiplicity fluctuations in a more convenient temperature range from 200 to 700 K to measure.

## 2. Samples and Experimental Methods

The rare-earth cobaltites NdCoO_3_ and SmCoO_3_ were obtained by conventional ceramic processing using a stoichiometric amount of oxides Co_3_O_4_, 99.7% (metals basis, Sigma-Aldrich, St. Louis, MO, USA), Nd_2_O_3_ and Sm_2_O_3_, 99.99% (Rare Metals Plant, Novosibirsk, Russia), which were thoroughly mixed and the resulting mixture was annealed at a temperature of 120ºC for 36 h with intermediate grinding. After annealing, the mixture was ground again, tablets were pressed in bars of 5 mm × 13 mm × 1 mm, which were then annealed in air at a temperature of 1200 °C for 8 h and cooled together with the furnace up to room temperature at 2 °C/min.

Powder X-ray diffraction (PXRD) data were collected on a PANalyticalX’Pert PRO powder diffractometer (Eindhoven, Netherlands) equipped with a solid- state detector using the CoK_α_-radiation in the range of 2θ = 20 –130° and within 300 K to 1000 K. The RCoO_3_ samples (R = Nd, Sm) were ground in octane in an agate mortar, dried and placed in a flat holder for PXRD measurements in the Bragg-Brentano geometry. X-ray investigations at high temperature were carried out in an Anton Paar HTK 1200N (AntonPaar, Austria) high-temperature chamber with sample rotation and self-adjustment. The samples were preliminarily kept in a high-temperature chamber for 2 h at a temperature of 1000K. The crystal lattice parameters were refined using the derivative difference minimization method (DDM) [40].

Static magnetization measurements in the temperature range from 1.8 to 400 K and magnetic field up to 50 000 Oe were carried out with a MPMS-XL Quantum Design SQUID magnetometer (USA).

Thermal expansion was studied in the temperature range 100–700 K with a Netzsch DIL-402C induction dilatometer in dynamic mode with heating and cooling rates of 3 K/min when purged with dry helium (O2 content ≈ 0.05% of the volume). The rod load on the sample is 30 sN. The fused silica standards to calibrate and account for the dilatation of measuring system were used.

The temperature dependences of the electrical resistance were obtained with the universal installation—Physical Properties Measurement System (PPMS-9) Quantum Design (USA) at the core facilities center of Lebedev Physical Institute of the Russian Academy of Sciences (Moscow).

## 3. Results and Discussions

### 3.1. X-Ray Phase and X-Ray Diffraction Analysis

**Figure 1:**
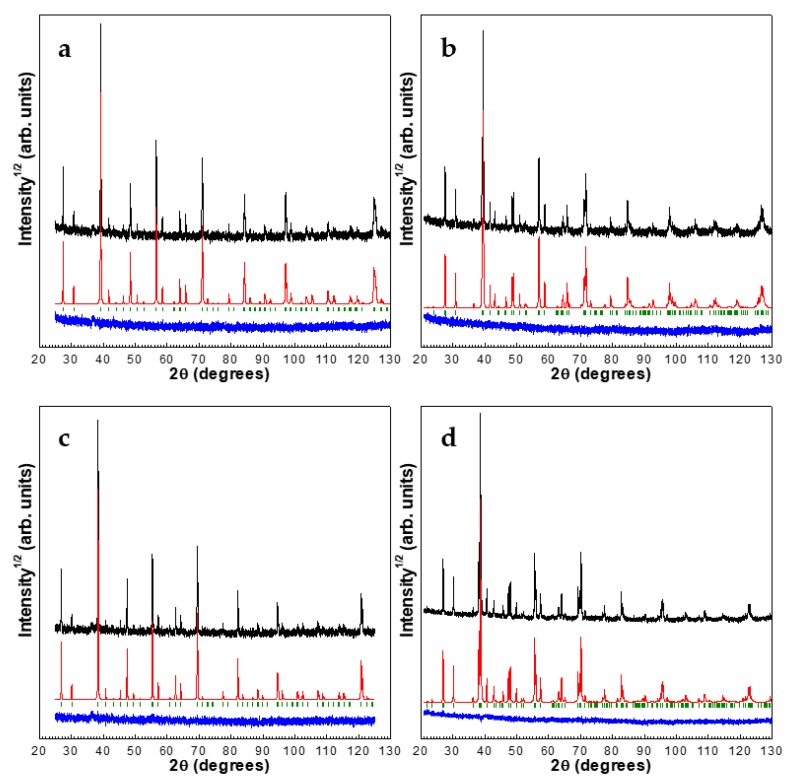
Experimental (upper, black), calculated (middle, red) and difference (lower, blue) Powder X-ray diffraction (PXRD) profiles after derivative difference minimization method (DDM) refinement; (**a**) NdCoO_3_ at 300 K, (**b**) SmCoO_3_ at 300 K, (**c**) NdCoO_3_ at 1000 K, and (**d**) SmCoO_3_ at 1000 K.

According to the PXRD analysis, the amount of cobalt oxide impurity in the samples was 1.5% and 2% for SmCoO_3_ and NdCoO_3_, respectively. Within the whole temperature range studied, the main phases have an orthorhombic perovskite-type structure with the *Pbnm*- space group. The experimental, calculated, and difference PXRD profiles after the DDM refinement at 300 K and 1000 K are presented in Figure 1.

The room temperature structural characteristics are consistent with other data available [41,42]. The crystal lattice parameters at various temperatures are summarized in Table 1. The deviation of the oxygen nonstoichiometry index from δ = 3, according to thermogravimetric analysis, does not exceed 0.6%.

**Figure 2:**
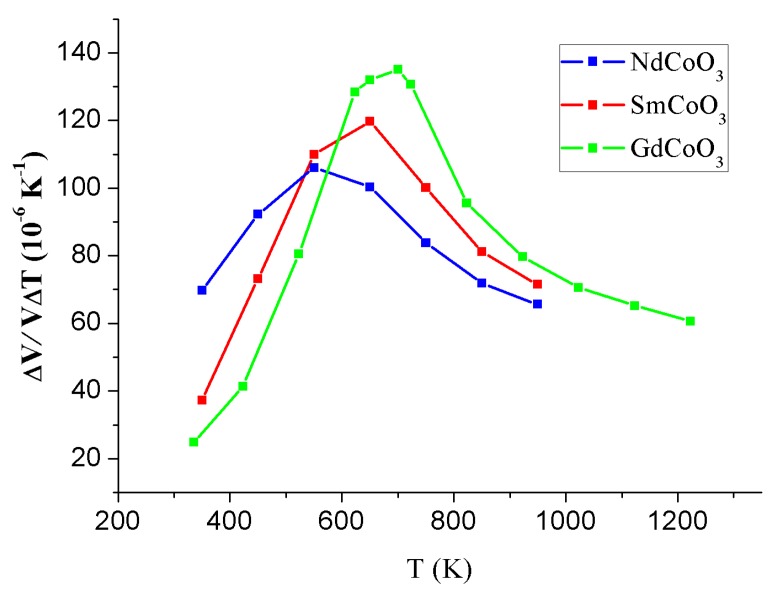
Temperature dependences of volume expansion coefficients for NdCoO_3_, SmCoO_3_, and GdCoO_3_. The data for GdCoO_3_ are taken from [39].

The temperature dependences of volume expansion coefficient for NdCoO_3_, SmCoO_3_, and GdCoO_3_ arepresented in Figure 2.

The given dependences are characterized by the presence of maxima within 550 K for NdCoO_3_ and 650 K for SmCoO_3_.

The combined analysis of the specific heat and thermal expansion of rare earth cobalt oxides and their solid solutions demonstrated that their temperature dependence exhibits characteristic anomalies related to the occupation of the high-spin states of cobalt ions and to the additional electron contribution arising at the insulator–metal transition occurring with the growth of the temperature. With the decrease in the radius of the rare earth ion or with the growth of chemical pressure, the spin gap in these compounds grows and is sample dependent, so we observed the shift of the low-temperature feature toward higher temperatures and the gradual merging of the two contributions to the specific heat and thermal expansion [43]. In Figure 2 the high-temperature feature of the thermal expansion of NdCoO_3_, SmCoO_3_, and GdCoO_3_ due to the insulator–metal transition observed for the entire series of rare-earth cobalt oxides with a characteristic transition temperature $T_{IM}$ increasing with decreasing ion radius of the rare-earth element is shown. $T_{IM}$ increasing determine the origin of the sample dependence in the volume expansion (Figure 2).

### 3.2. Magnetic Properties

**Figure 3:**
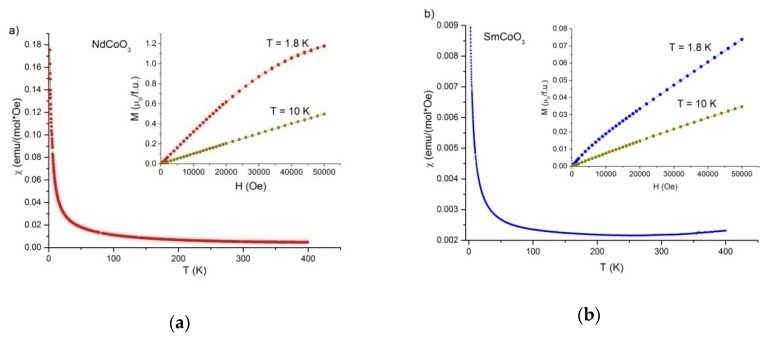
Temperature dependences of the molar magnetic susceptibility for NdCoO_3_ (**a**) and SmCoO_3_ (**b**) samples (H = 15 000 Oe). The insets show the magnetization curves at T = 1.8 and 10 K.

The temperature dependences of the molar magnetic susceptibility χ(T) and field dependences of the magnetic moment M(H) of the NdCoO_3_ and SmCoO_3_ samples are shown in Figure 3. The magnetic moment values of NdCoO_3_ are almost fifteen times higher than the similar values for SmCoO_3_. The magnetization curves obtained in the FC and ZFC modes do not differ from each other for both samples. In contrast to the behavior of the NdCoO_3_ magnetic susceptibility, being reduced progressively with increasing temperature in the entire studied range (Figure 3a), the SmCoO_3_ susceptibility is characterized by a plateau in the temperature range 180–270 K. A further temperature raise leads to an increase in the SmCoO_3_magnetic susceptibility (Figure 3b) which is caused by the appearance of a contribution from Co^3+^ ions at high temperatures. The temperature dependence of the magnetic susceptibility of RCoO_3_ is determined by the magnetization of rare-earth ions and the additional paramagnetic contribution induced by the thermally excited magnetic terms of Co^3+^ ions. The obtained experimental data are in good agreement with similar studies in recently published works [44,45].

The field dependences of magnetization correspond to paramagnetic behavior (Figure 3, insets), while maintaining the linearity in the region of weak fields. The magnetization saturation trends at T = 1.8 K are not observed in the entire field range up to 50 000 Oe for both samples.

**Figure 4:**
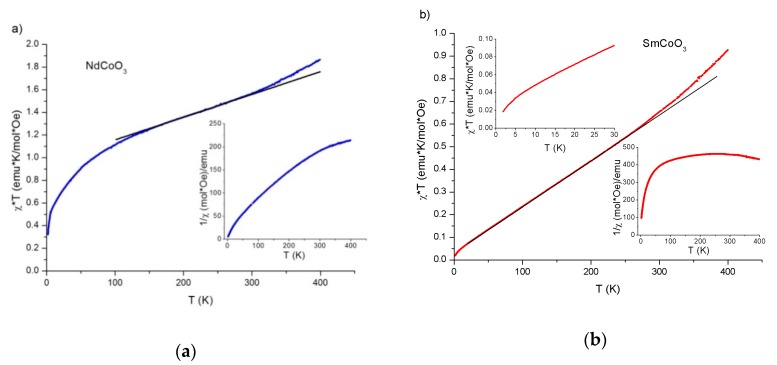
Temperature dependences of the reduced magnetic susceptibility χT for NdCoO_3_ (**a**) and SmCoO_3_ (**b**) samples. The temperature dependences of the inverse magnetic susceptibility (NdCoO_3_ (**a**), SmCoO_3_ (**b**)) and the χT dependence for SmCoO_3_ in the low-temperature region (**b**) are presented in the insets.

The temperature dependences of the inverse molar susceptibility 1/χ of the NdCoO_3_ samples (Figure 4a, inset) and SmCoO_3_ (Figure 4b, inset) are shown in Figure 4. In the entire temperature range under consideration, these dependences do not obey the Curie–Weiss law. Taking into account the linearity of temperature dependence of the reduced magnetic susceptibility χT in a certain temperature range (Figure 4), it is worth describing the magnetic sample properties in order to divide the temperature range into a low-temperature, high-temperature, and intermediate interval where dependence is linear.

The χT dependences in the intermediate temperature range were approximated by straight lines with convergence coefficients R equal to 0.99999 for SmCoO_3_ and 0.99913 for NdCoO_3_. The linearity of the χT dependence allows one to describe the magnetic susceptibility in the interval as a superposition of two contributions: $\chi =C/T+\chi_{VV}$, where C/T is the orientation paramagnetic Curie susceptibility of rare-earth ions, and $\chi_{VV}$ is the Van Vleck polarization susceptibility. The diamagnetic contribution of the electron shells is sufficiently small, and therefore is ignored.

The temperature ranges, the calculated values C, $\chi_{VV}$ obtained by experiment and effective magnetic moments $\mu_{eff}^{\exp}$ for NdCoO_3_ and SmCoO_3_ compounds in the intermediate temperature range $T_{\min}$–$T_{\max}$ are shown in Table 2. Some theoretical values are also given [46].

The obtained values of C and $\mu_{eff}^{\exp}$ are a little larger than the similar values obtained in [47] for SmCoO_3_ (C = 0.0276 emu·K/(mol·Oe), $\mu_{eff}$ = 0.47 µ_B_). The effective magnetic moments for both Nd^3+^ and Sm^3+^ are significantly lower than their theoretical values calculated for free ions (Table 2).

**Figure 5:**
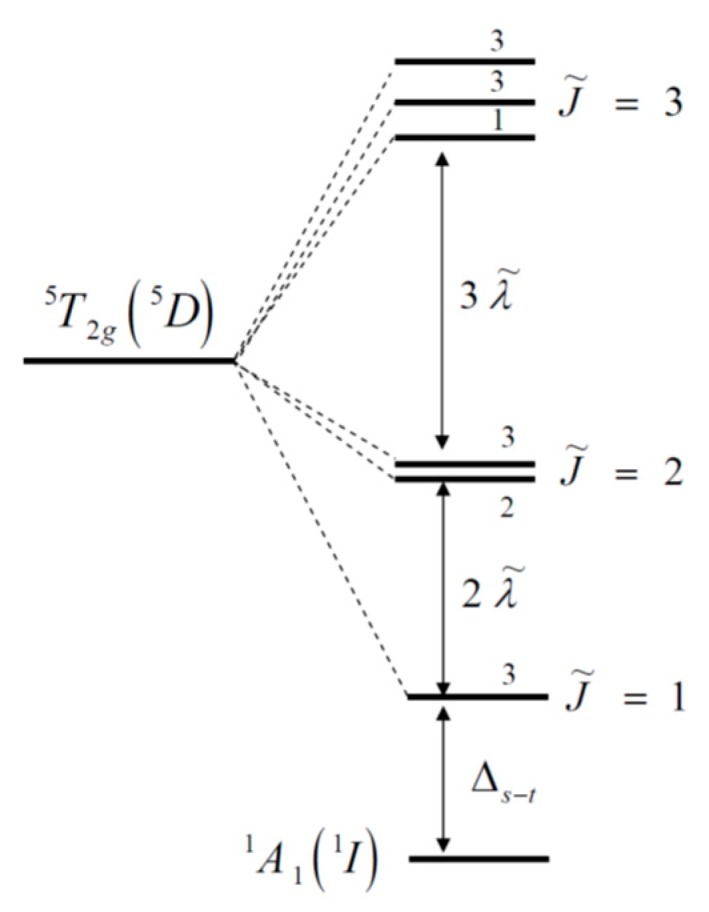
A set of low-energy terms for the d^6^ electronic configuration for Co^3+^ ion in a crystalline field of octahedral symmetry, taking into account spin–orbit interaction. The degeneracy multiplicity is shown by digits for terms [15].

The total magnetic susceptibility of NdCoO_3_ and SmCoO_3_ can be represented as a sum of two independent summands (since the Co ions acquire a magnetic moment only at high temperatures, the exchange interaction of Co-RE can be neglected)

(1) $$\chi_{Sm\left(Nd\right)CoO_{3}}=\chi_{Sm\left(Nd\right)}+\chi_{Co}$$

where $\chi_{Sm\left(Nd\right)}$ and $\chi_{Co}$ are the magnetic susceptibilities of samarium (neodymium) and cobalt ions, respectively. To describe the contribution of cobalt ions to the total magnetization of Sm(Nd)CoO_3_, a diagram of the Co^3+^ ion levels in a crystal field taking into account the spin–orbit interaction is shown in Figure 5. The ground term is a A11 low-spin singlet, separated from the triplet sublevel $\tilde{J}=1$ of the T25 high-spin state by the $\Delta_{S}$ spin gap. At $\Delta_{S}=150$ K, the position of the terms for LaCoO_3_corresponds to [13,23,48]. Lanthanum substitution on another rare-earth ion with a smaller ionic radius leads to a chemical pressure generation that is equivalent to the external pressure. Therefore, the substitution will lead to the additional stabilization of low-spin state or, in other words, to increase the spin gap.

At low temperatures, there are the SmCoO_3_ and NdCoO_3_ cobalt ions in the A11 nonmagnetic low-spin state. With increasing temperature, thermal excitations of the high-spin state with a nonzero magnetic moment (the multiplicity fluctuations) and increase of the magnetization occur. The statistical sum of the Co^3+^ ions per mole of substance can be represented as:

(2) $$Z=[1+e^{-\beta \Delta_{S}}+2e^{-\beta \Delta_{S}}ch\left(g_{1}\mu_{B}\tilde{B}\beta \right)+e^{-\beta \left(\Delta_{S}+2{\tilde{\lambda }}_{Co}\right)}+2e^{-\beta \left(\Delta_{S}+2{\tilde{\lambda }}_{Co}\right)}ch\left({g^{\prime }}_{2}\mu_{B}\tilde{B}\beta \right)\;{+2e^{-\beta \left(\Delta_{S}+2{\tilde{\lambda }}_{Co}\right)}ch\left({g^{\prime \prime }}_{2}\mu_{B}\tilde{B}\beta \right)]}^{N_{A}}$$

where ${\tilde{\lambda }}_{Co}=185$ K [48] is the effective constant of spin–orbit interaction, $N_{A}$ is the Avogadro number, $\tilde{B}$ is the applied external magnetic field, $k_{B}$ is the Boltzmann constant, $\beta =1/k_{B}T$, $\mu_{B}$ is the Bohr magneton, the Landé factors $g_{1}=3.4$ for the triplet $\tilde{J}=1$ and ${g^{\prime }}_{2}=3.1$, ${g^{\prime \prime }}_{2}=1.8$ for the quintet $\tilde{J}=1$. With the partition function, the free energy $F=-k_{B}T\ln Z$ and magnetization $M=-\partial F/\partial \tilde{B}$ are found in a standard way. For not too low temperatures and not too strong magnetic fields, the expression for the molar magnetic susceptibility of Co^3+^ ions $\chi_{Co}=\partial M/\partial \tilde{B}$ is as follows:

(3) $$\chi_{Co}=N_{A}2\mu_{B}^{2}\beta \left[g_{1}^{2}e^{-\beta \Delta_{S}}+{g^{\prime }}^{2}e^{-\beta \left(\Delta_{S}+2\tilde{\lambda }\right)}+{g^{\prime \prime }}^{2}e^{-\beta \left(\Delta_{S}+2\tilde{\lambda }\right)}\right]/\left[1+3e^{-\beta \Delta_{S}}+5e^{-\beta \left(\Delta_{S}+2\tilde{\lambda }\right)}\right]$$

In the case when $\Delta_{S}>1000$ K, the spin–orbit interaction can be neglected, the expression (3) takes the form:

(4) $$\chi_{Co}=N_{A}\frac{g^{2}\mu_{B}^{2}S\left(S+1\right)}{3k_{B}T}n_{HS}$$

where g=2 is the Lande spin factor, $n_{HS}=\frac{g_{HS}\exp\left(-\Delta_{S}/k_{B}T\right)}{1+g_{HS}\exp\left(-\Delta_{S}/k_{B}T\right)}$ is the population of the HS- state, $g_{HS}=\left(2S+1\right)\left(2L+1\right)=15$ for the high-spin state with S=2, L=1.

**Figure 6:**
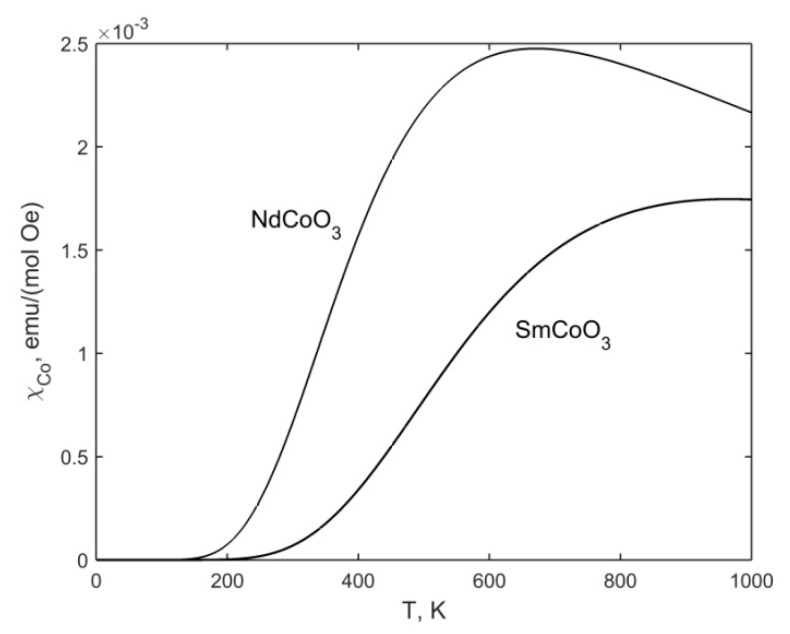
Temperature dependences of the Co^3+^ ion magnetic susceptibility for NdCoO_3_ and SmCoO_3_ samples.

The calculation results $\chi_{Co}$ for NdCoO_3_ and SmCoO_3_are given in Figure 6. The following values are used: $g_{HS}=15$, $\Delta_{S}=$ 2300 K (SmCoO_3_) and $\Delta_{S}=$ 1600 K (NdCoO_3_).

The magnetic susceptibility of Sm^3+^ ions in SmCoO_3_can be represented by the formula [49].

(5) $$\chi_{Sm}=\frac{0.2482}{xT}\frac{1.07x+3.67+\left(21.45x+0.82\right)e^{-7x/2}+\ldots }{3+4e^{-7x/2}+\ldots }$$

where $x=\lambda_{Sm}/T$, $\lambda_{Sm}$ is the spin–orbit coupling constant of the rare-earth Sm^3+^ ion. It is known from spectroscopic data that the nearest excited term ^6^H_7/2_ of the Sm^3+^ ion is separated from the main one by the energy interval $\Delta =7/2\lambda_{Sm}$ approximately 1000 cm^−1^ [49], therefore $\lambda_{Sm}\approx$ 400 K. Despite the fact that formula (5) was obtained for a free rare-earth ion with no crystalline field, however, it is possible to describe the temperature dependence of the SmCoO_3_ magnetic susceptibility as will be seen below.

Within low temperatures, the expression (5) takes a simpler form:

(6) $$\chi_{Sm}\approx C/T+\chi_{VV}$$

where C is the effective Curie constant, and $\chi_{VV}$ is the Van Vleck susceptibility.

**Figure 7:**
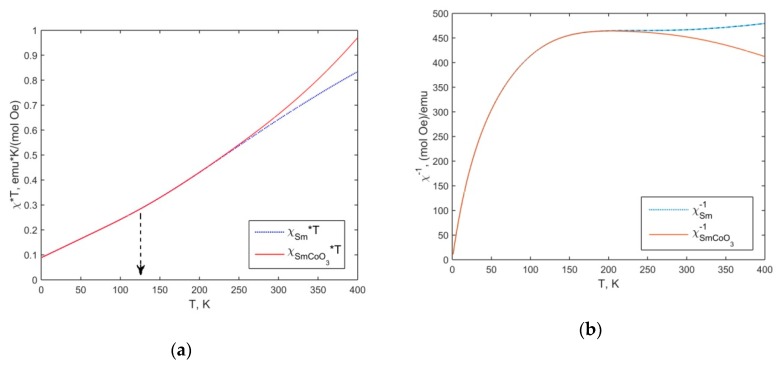
The calculation results of the temperature dependences χT (**a**) and $\chi^{-1}$ (**b**), respectively, for SmCoO_3_ (red solid line). By contrast, the contribution of only Sm^3+^ ions is shown by blue dashed line.

The calculation results χT and $\chi^{-1}$ respectively, for SmCoO_3_ (red solid line), using (1), taking into account (4) and (5) are presented in Figure 7a,b. In contrast, in Figure 7, the contribution of only Sm^3+^ions, according to (5) is depicted by the blue dashed line. That can be seen to be in a good agreement with the experiment. The theoretical dependence χT (Figure 7a) shows a smooth deviation from the linear dependence at T ≈ 125 K (shown by the dashed arrow), below this temperature the expression (6) is valid. Nevertheless, the approximation (6) for SmCoO_3_can be seen from Figure 4b to be valid over a wide temperature range up to 250 K(see above). Thus, Co^3+^ magnetic moment is provided above 250K for SmCoO_3_ that agrees with Figure 6.

The ground state of free Nd^3+^ ion with the 4*f*^3^ electronic configuration is the ^4^I_9/2_ multiplet (L = 6, S = 3/2). The nearest excited state ^4^I_11/2_ is 1900 cm^−1^higher in energy. As for the free Sm^3+^ ion, its electronic configuration is 4*f*^5^, the ground multiplet state is ^6^H_5/2_ (L = 5, S = 5/2). The distinctive feature of this ion is the relative proximity to the first excited state ^6^H_7/2_. The energy difference of these states for free Sm^3+^ ion is approximately 1000 cm^−1^ [49].

The magnetic properties for ions essentially depend on their environment, i.e., on the crystal field value and symmetry. Hence, in perovskite-like crystals, such as cobaltites, the rare-earth ion is in a low-symmetrical ligand environment. The field of such symmetry splits the main Sm^3+^ multiplet into three and five Kramers doublets for Nd^3+^, with each of them having a certain magnetic moment. In the general case, such a splitting leads to a decrease or, by contrast, an increase in the magnetic ion moment and, in addition, the influence of the crystal field can be expressed in a significant difference between the g- factor of Kramers doublets and $g_{0}=\frac{3}{2}-\frac{L\left(L+1\right)-S\left(S+1\right)}{2J\left(J+1\right)}$ for the free ion and their strong anisotropy, which turns to result in magnetic susceptibility anisotropy. For polycrystalline samples, the average magnetic susceptibility can be calculated as $\langle \chi \rangle =(\chi_{∥}+2\chi_{⊥})/3$, where $\chi_{∥}$ and $\chi_{⊥}$ are the susceptibility components in parallel and perpendicular directions to the external applied magnetic field. Therefore, the complex energy level structure of the Sm^3+^ and Nd^3+^ ions in a crystal field of low symmetry leads to the temperature dependence in the low-temperature region ($T<T_{\min}$) shown in Figure 4.

It has been known that in order to calculate the temperature dependence of magnetization and susceptibility, the positions of the energy levels of the $E_{n}$ system taking into account the external magnetic field have to be realized. Van Fleck (1932) studied the energy contributions in terms ofperturbative approach depending on the effect of the magnetic field H: $E_{n}=E_{n}^{\left(0\right)}+HE_{n}^{\left(1\right)}+H^{2}E_{n}^{\left(2\right)}$, where $E_{n}^{\left(0\right)}$ are the energy system levels without the external magnetic field ${\hat{H}}_{0}|n\rangle =E_{n}^{\left(0\right)}|n\rangle$, generally developing the groups of degenerate states; $E_{n}^{\left(1\right)}=\langle n\left|\mu_{B}\left({\hat{L}}_{s}+g_{0}{\hat{S}}_{s}\right)\right|n\rangle$, $E_{n}^{\left(2\right)}=\sum_{n^{\prime }\neq n}\frac{{\left|\langle n\left|\mu_{B}\left({\hat{L}}_{s}+g_{0}{\hat{S}}_{s}\right)\right|n^{\prime }\rangle \right|}^{2}}{E_{n}-E_{n}^{\prime }}$ are Zeeman coefficients of the first and second order (the z axis is directed along the magnetic field). The Hamiltonian ${\hat{H}}_{0}$ contains inter-electron repulsion, spin–orbit interaction, and crystal field energy.

The temperature dependence equation of magnetic susceptibility, known as the Van Vleck equation [50], has the form: $\chi_{Vleck}=N_{A}\frac{\sum_{n}\left[\frac{{\left(E_{n}^{\left(1\right)}\right)}^{2}}{k_{B}T}-2E_{n}^{\left(2\right)}\right]\exp\left(-\frac{E_{n}^{\left(0\right)}}{k_{B}T}\right)}{\sum_{n}\exp\left(-\frac{E_{n}^{\left(0\right)}}{k_{B}T}\right)}$, where $N_{A}$ is the Avogadro constant, and $k_{B}$ is the Boltzmann constant. It is generally accepted that, in commonly used H≤10 kOe fields, the Zeeman interaction energy is usually less than the splitting caused by inter-electron repulsion, crystal field, and spin–orbit interaction; however, in case of rare-earth ions in low-symmetry crystal fields, the multiplet splitting into Kramers sublevels being sufficiently close to each other (see above) seems to be compared with the interaction energy with magnetic field and to be observed by an unusual dependence of paramagnetic Van Vleck susceptibility on the magnetic field value. Thus, to characterize properly the magnetic properties of the rare-earth and transition metal ions in low-symmetry fields, both the low-symmetry part of crystal field and the magnetic field influence have to be similarly considered or, in other words, simultaneously taken into account.

In contrast to SmCoO_3_, NdCoO_3_ has a linear region χT and $\chi^{-1}$ in a more narrow temperature range (see Table 2). Above $T_{\max}$ ≈ 250 K, the contribution of Co^3+^ ions to the total magnetic susceptibility of the sample becomes detectible for NdCoO_3_.

In a low-symmetry crystal field, the main term ^6^H_5/2_ of the Sm^3+^ ion splits into three Kramers doublets, and the main term ^4^I_9/2_ of the Nd^3+^ ion splits into five ones in a wider energy range [49]. This causes a significant difference in the $T_{\min}$ temperature for SmCoO_3_ and NdCoO_3_ (see Table 2). As otherwise stated, with decreasing temperature for NdCoO_3_, crystal field effects are important even at $T_{\min}$ ≈ 165 K, while for SmCoO_3_ only at $T<T_{\min}$ ≈ 15K.

The origin of Van Vleck paramagnetism is to add the wave functions of thermally unpopulated excited states to the wave functions of ground state. The Van Vleck susceptibility of the free Sm^3+^ (Nd^3+^) ions is due to possible (virtual) quantum transitions between the energetically lowest ^6^H_5/2_ (^4^I_9/2_) state and the nearest excited ^6^H_7/2_ (^4^I_11/2_) state. In the crystal field, additional multiplet splitting occurs and, besides the indicated transitions, some possible ones within the same multiplet have to be taken into account. This is the reason for the difference between the Van Vleck susceptibility of SmCoO_3_ and NdCoO_3_ and that for free ions.

The electronic structure of cobaltites calculated in the framework of the LDA + GTB multi-electron approach [17,39] depends on the $n_{HS}$ concentration. Therefore, correlation of the changes in activation energy with changes in thermal expansion and magnetic susceptibility is also due to the contribution of an increasing concentration of high-spin states with temperature rise.

### 3.3. Thermal Expansion

**Figure 8:**
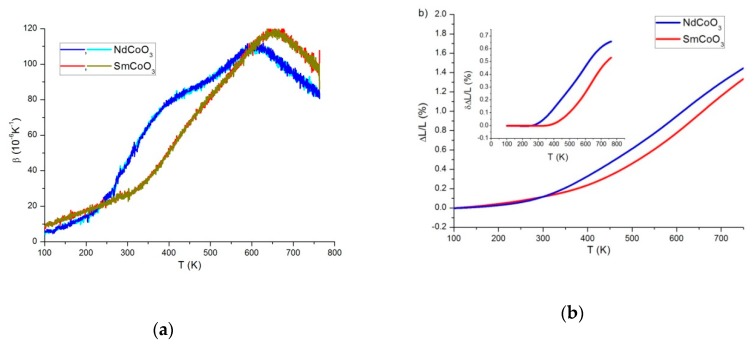
Temperature dependences of the volume thermal expansion coefficient β, obtained as a result of successive heating–cooling cycles (**a**) and (ΔL/L) deformation (**b**), for NdCoO_3_ and SmCoO_3_ samples. The inset shows abnormal contributions to the deformation after subtraction of the standard linear contribution to the lattice expansion.

The experimental temperature dependences of the volume thermal expansion coefficient β(T), obtained in the heating and cooling modes are presented in Figure 8. Hysteretic phenomena were not observed. The coefficient β for NdCoO_3_ compound is characterized by the presence of two diffuse anomalies near 400 and 600 K, and for SmCoO_3_by one maximum near 650 K (Figure 8a). Deviation from the usual linear contribution due to anharmonicity occurs in the temperature ranges of 250–270 K and 310–330 K for NdCoO_3_ and SmCoO_3_, observed in the temperature dependences of the deformation ΔL/L (Figure 8b). In this case, the temperatures of the first maximum for NdCoO_3_ and the maximum for SmCoO_3_ correlate with the maxima obtained on the temperature dependences of the thermal expansion during diffraction studies. The anomalous contribution of electronic origin due to multiplicity fluctuations is revealed by the deviations from linear behavior shown in the inset to Figure 8.

In the case of spin crossover materials, a large contribution to the anomaly of thermal expansion is made by the redistribution of the HS/LS statistical weights due to the large difference in their ionic radii [39]; therefore, the unit cell volume as a temperature function can be represented as

(7) $$V\left(T\right)=V_{HS}\left(T\right)n_{HS}\left(T\right)+V_{LS}\left(T\right)n_{LS}\left(T\right)$$

where, $V_{HS}\left(T\right)$, $V_{LS}\left(T\right)$ is the unit cell volume, respectively, in the phase of the HS- and LS- states, $n_{HS/LS}\left(T\right)$ is the population of HS/LS- states., this turns to be represented as

(8) $$V_{HS}\left(T\right)=V_{HS}^{\left(0\right)}\left(1+\beta_{HS}T\right)$$

and

(9) $$V_{LS}\left(T\right)=V_{LS}^{\left(0\right)}\left(1+\beta_{LS}T\right)$$

where $\beta_{HS/LS}$ is the volumetric thermal expansion coefficient, and $V_{HS/LS}^{\left(0\right)}$ is the unit cell volume when T = 0, respectively, in the phase of the HS/LS- state. In the case of rare-earth cobalt oxides, the non-magnetic LS- state is the ground state of cobalt ion, and the HS-state is possible with increasing temperature, therefore expression (8) is suggested to be written by the so-called «virtual crystal model», when the ground state of cobalt ions is the artificially created HS-state (hypothetical HS-phase). A similar approach was previously used to describe the thermodynamic and magnetic properties of GdCoO_3_ [39], where $V_{HS/LS}^{\left(0\right)}$ was determined by first-principle calculations using the DFT method. Since $n_{LS}\left(T\right)=1-n_{HS}\left(T\right)$, then

(10) $$V\left(T\right)=\left(V_{HS}^{\left(0\right)}-V_{LS}^{\left(0\right)}\right)n_{HS}\left(T\right)+\left(V_{HS}^{\left(0\right)}\beta_{HS}-V_{LS}^{\left(0\right)}\beta_{LS}\right)Tn_{HS}\left(T\right)+V_{LS}^{\left(0\right)}\left(1+\beta_{LS}T\right)$$

**Figure 9:**
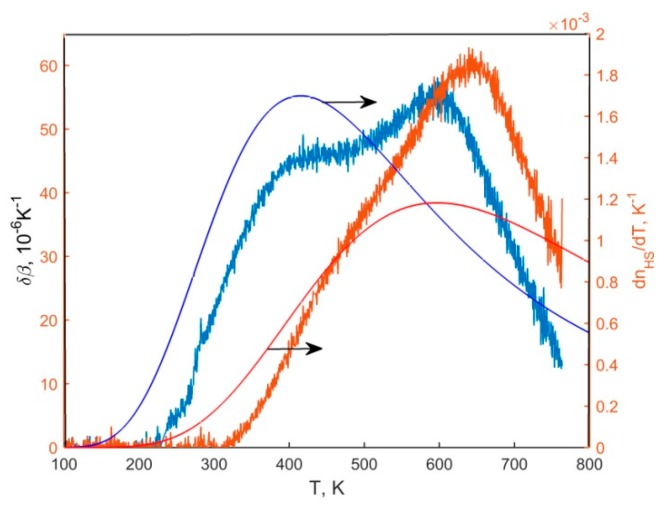
Anomalous contributions to the volumetric thermal expansion coefficient for NdCoO_3_ (depicted by blue color) and SmCoO_3_ (depicted by red color). Solid blue and red lines show the calculated dependences $\partial n_{HS}/\partial T$ for NdCoO_3_ and SmCoO_3_, respectively.

In expression (10), both the background (regular) contribution, i.e., the second and third summands due to the anharmonicity of lattice vibrations in the phase of mixed LS/HS- and pure LS- states, and the anomalous contribution of thermal expansion, i.e., the first summand arising due to multiplicity fluctuations of cobalt ions can be distinguished. Since the characteristic values are $\beta_{HS/LS}∼10^{-5}$ 1/K, and $0\leq n_{HS/LS}\left(T\right)<1$ then at T<1000 K, the first summand makes the largest contribution compared to the second one, therefore, the volumetric thermal expansion coefficient can be represented as $\beta =\frac{1}{V}\frac{\partial V}{\partial T}\approx \delta \beta +\beta_{reg}$, where $\delta \beta =\frac{\left(V_{HS}^{\left(0\right)}-V_{LS}^{\left(0\right)}\right)}{V_{LS}^{\left(0\right)}}\frac{\partial n_{HS}\left(T\right)}{\partial T}$. Thus, the anomalous contribution to the volumetric thermal expansion coefficient is proportional to the first-order derivative with respect to the population temperature of the HS- state $n_{HS}$ and is determined by the magnitude of spin gap. In Figure 9, by comparison, the experimental data of anomalous contribution to thermal expansion and the calculated values $\partial n_{HS}/\partial T$ for NdCoO_3_ and SmCoO_3_ at $\Delta_{S}=$1600 and 2300 K, respectively, are presented.

Figure 8a and Figure 9 illustrate that, in contrast to SmCoO_3_, in temperature dependence of the volumetric thermal expansion coefficient of NdCoO_3_, two maxima can be clearly distinguished. The first (low-temperature) maximum is associated with fluctuations of the spin multiplicity of cobalt ions, and the second one with the insulator–semimetal transition (crossover) observed for all rare-earth cobalt oxides with increasing temperature and characteristic transition temperature of the rare-earth element. For SmCoO_3_, the spin gap obtained above by the magnetic data analysis is large enough, thus both peaks almost coincide. A similar situation is observed for solid solutions of rare-earth cobaltites. Then, in La_1-*x*_Gd*_x_*CoO_3_, the low-temperature maximum of thermal expansion shifts to the region of higher temperatures with an increase in the gadolinium concentration and gradually coincides with the second maximum [43].

### 3.4. Transport Properties

**Figure 10:**
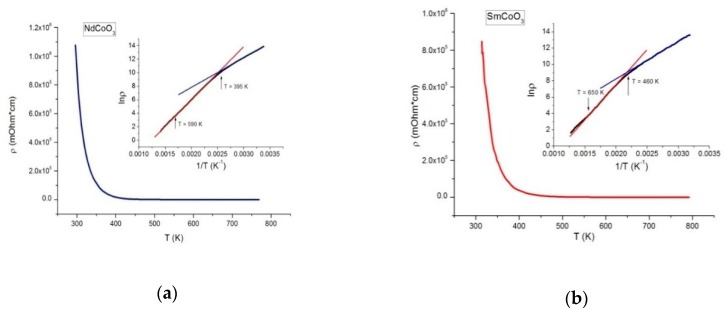
Temperature dependence of electrical resistivity for the NdCoO_3_ (**a**) and SmCoO_3_ (**b**) samples. The corresponding dependencies of the resistivity logarithm on the reciprocal temperature are illustrated in the insets. Straight lines show accordance with the thermo-activation law (blue—the region of intermediate temperatures, red—high temperatures).

The temperature dependences of the electrical resistivity ρ(T) for NdCoO_3_ and SmCoO_3_ samples and the dependence of resistivity logarithm on the reciprocal temperature are shown in Figure 10. The ρ(T) dependences reliably correspond to the semiconductor type dρ(T)/dT<0 over the studied 300 to 750 K range. According to the lnρ(1/T) dependences, it is matter of fact that the semiconductor type of conductivity can be described using the currently accepted thermal activation relation of the form $\rho (T)=\rho_{\infty }\exp(E_{a}/k_{B}T)$, where $E_{a}$ is the activation energy [51], and $\rho_{\infty }$ is the constant determined by T→∞. Moreover, for each sample, there is a temperature $T^{*}$ when the activation energy changes in the intermediate region and temperature $T^{**}$ characterizing the variation from the activation law at high temperatures. The values of the obtained parameters are presented in Table 3.

Accordance with the thermal activation law is depicted by straight lines in the insets to Figure 10.

The temperature deviations from a thermal activation law and the characteristic temperatures of changes in the activation energy correlate with anomalies in the temperature dependences of the volumetric thermal expansion coefficient β(T) for both samples.

## 4. Conclusions

The features of thermal expansion, magnetic susceptibility, and transport properties of NdCoO_3_ and SmCoO_3_ cobaltites being in a good agreement were experimentally demonstrated. Partially, these correlations were previously known, e.g., those of thermal expansion with a spin and electronic transition [5]. The features have been shown theoretically to be associated with a population increase of high-spin states of Co^3+^ ions. A comparison of the results with well- studied GdCoO_3_ allows one to identify both general trends inherent in all rare-earth cobaltites based on lanthanide compression and the specific properties of samples containingNd^3+^, Sm^3+^ions formed with strong single-ion anisotropy and crystal field effects at low temperatures.

A quantitative assessment of the contribution from the fluctuations of multiplicity to the magnetic properties of NdCoO_3_ and SmCoO_3_ samples seems to be a rather difficult task, since in addition to the complex structure of the energy levels of Sm^3+^ and Nd^3+^ ions in a low-symmetry crystal field, it is necessary to take into account the influence of oxygen non-stoichiometry of the samples referred to a number of research works [52,53]. On the one hand, oxygen non-stoichiometry is the main reason for defects in the structure of rare-earth cobaltites leading to the formation of magnetic excitons [54,55]; on the other hand, it can lead to dimer formation [56] and the appearance of Co^3+^ ions in the HS- state even at low temperatures.

## Figures and Tables

**Table 1:** Crystal lattice parameters for NdCoO_3_ and SmCoO_3_ at various temperatures.

	NdCoO_3_				SmCoO_3_			
T, K	*a*, Å	*b*, Å	*c*, Å	V, Å^3^	*a*, Å	*b*, Å	*c*, Å	V, Å^3^
300	5.3478(1)	5.3324(1)	7.5505(2)	215.32(1)	5.2887(1)	5.3517(3)	7.5031(2)	212.37(1)
400	5.3591(1)	5.3463(2)	7.5677(2)	216.82(1)	5.2961(1)	5.3572(1)	7.5130(2)	213.16(1)
500	5.3733(1)	5.3649(1)	7.5909(3)	218.82(1)	5.3082(1)	5.3726(1)	7.5291(1)	214.72(1)
600	5.3907(2)	5.3888(1)	7.6176(2)	221.29(1)	5.3246(1)	5.3985(1)	7.5521(1)	217.08(1)
700	5.4067(1)	5.4106(1)	7.6427(3)	223.57(1)	5.3423(1)	5.4292(1)	7.5777(1)	219.78(1)
800	5.4208(1)	5.4270(1)	7.6635(1)	225.45(1)	5.3573(1)	5.4519(1)	7.6001(1)	221.98(1)
900	5.4332(1)	5.4404(1)	7.6819(2)	227.07(1)	5.3707(1)	5.4683(1)	7.6197(1)	223.78(1)
1000	5.4451(1)	5.4521(2)	7.6989(3)	228.56(1)	5.3831(1)	5.4816(1)	7.6380(1)	225.38(1)

**Table 2:** and $T_{\max}$ are boundaries of the temperature range, where the χT dependence is linear, C is the Curie constant of orientation paramagnetic susceptibility, $\chi_{VV}$ is the Van Vleck polarization susceptibility, $\mu_{eff}^{\exp}$ is the value of the experimentally obtained effective magnetic moment, $\mu_{eff}^{\mathrm{teor}}$ is the theoretical value of the effective magnetic moment, $\mu_{eff}^{\mathrm{teor}(\mathrm{VV})}$ is the theoretical value of the effective magnetic moment taking into account the Van Vleck paramagnetism, R is the convergence coefficient of the experimental data and the fitting line in the given temperature range.

	$T_{\min}$ (K)	$T_{\max}$ (K)	$C\left(\frac{emu\cdot K}{mol\cdot Oe}\right)$	$\chi_{VV}\left(\frac{emu}{mol\cdot Oe}\right)$	$\mu_{eff}^{\exp}$ µ_B_	$\mu_{eff}^{\mathrm{teor}}$ µ_B_	$\mu_{eff}^{\mathrm{teor}(\mathrm{VV})}$ µ_B_	R
NdCoO_3_	165	250	0.956	0.00201	2.77	3.62	3.68	0.99993
SmCoO_3_	15	270	0.03504	0.00202	0.53	0.84	1.55	0.99999

**Table 3:** The parameters describing the thermal activation conductivity of the samples and the temperatures of NdCoO_3_ and SmCoO_3_ electronic transition (R is the convergence coefficient).

	T^*^, K	$T<T^{*}$			$T>T^{*}$			T^**^, K
		$E_{a}$, eV	$\rho_{\infty }$, mOhm·cm	R	$E_{a}$, eV	$\rho_{\infty }$, mOhm·cm	R	
NdCoO_3_	395	0.379 ± 0.001	0.390 ± 0.001	0.99955	0.679 ± 0.001	5.81 × 10^−5^	0.99991	590
SmCoO_3_	460	0.394 ± 0.001	0.401 ± 0.001	0.99893	0.739 ± 0.001	6.56 × 10^−5^	0.99944	650

## References

[1] Goodenough J.B. (1958). An interpretation of the magnetic properties of the perovskite-type mixed crystals La_1− x_Sr_x_CoO_3−δ_. J. Phys. Chem. Solids.

[2] Raccah P.M., Goodenough J.B. (1967). First-order localized-electron ⇆ Collective-electron transition in LaCoO_3_. Phys. Rev..

[3] Bhide V.G., Rajoria D.S. (1972). Mössbauer studies of the high-spin—Low-spin equilibria and the localized-collective electron transition in LaCoO_3_. Phys. Rev. B.

[4] Asai K., Yokokura O., Nishimori N., Chou H., Tranquada J.M., Shirane G., Higuchi S., Okajima Y., Kohn K. (1994). Neutron-scattering study of the spin-state transition and magnetic correlations in La_1−*x*_Sr*_x_*CoO_3_ (*x* = 0 and 0.08). Phys. Rev. B.

[5] Knížek K., Jirak Z., Hejtmanek J., Veverka M., Marysko M., Maris G., Palstra T.T.M. (2005). Structural anomalies associated with the electronic and spin transitions in LnCoO_3_. Eur. Phys. J. B Condens. Matter Complex Syst..

[6] Alonso J.A., Martinez-Lope M.J., de la Calle C., Pomjakushin V. (2006). Preparation and structural study from neutron diffraction data of RCoO_3_ (R = Pr, Tb, Dy, Ho, Er, Tm, Yb, Lu) perovskites. J. Mater. Chem..

[7] Berggold K., Kriener M., Becker P., Benomar M., Reuther M., Zobel C., Lorenz T. (2008). Anomalous expansion and phonon damping due to the Co spin-state transition in RCoO_3_ (R = La, Pr, Nd, and Eu). Phys. Rev. B.

[8] Vonsovskii S.V., Svirskii M.S. (1965). *ZhETF***1965**, *47*, 1354. J. Exp. Theor. Phys..

[9] Lyubutin I.S., Struzhkin V.V., Mironovich A.A., Gavriliuk A.G., Naumov P.G., Lin J.F., Ovchinnikov S.G., Sinogeikin S., Chow P., Xiao Y. (2013). Quantum critical point and spin fluctuations in lower mantle ferropericlase. Proc. Natl. Acad. Sci. USA.

[10] Jeong D.W., Choi W.S., Okamoto S., Kim J.-Y., Kim K.W., Moon S.J., Cho D.-Y., Lee H.N., Noh T.W. (2014). Dimensionality control of d-orbital occupation in oxide superlattices. Sci. Rep..

[11] Ivanova N.B., Ovchinnikov S.G., Korshunov M.M., Eremin I.M., Kazak N.V. (2009). Specific features of spin, charge, and orbital ordering in cobaltites. Phys. Uspekhi.

[12] Bernard R., Seikh M. (2012). Cobalt Oxides: From Crystal Chemistry to Physics.

[13] Noguchi S., Kawamata S., Okuda K., Nojiri H., Motokawa M. (2002). Evidence for the excited triplet of Co^3+^ in LaCoO_3_. Phys. Rev. B.

[14] Haverkort M.W., Hu Z., Cezar J.C., Burnus T., Hartmann H., Reuther M., Zobel C., Lorenz T., Tanaka A., Brookes N.B. (2006). Spin state transition in LaCoO_3_ studied using soft X-ray absorption spectroscopy and magnetic circular dichroism. Phys. Rev. Lett..

[15] Ropka Z., Radwanski R.J. (2002). The Jahn–Teller-effect formation of the non-magnetic state of the Co^3+^ ion in LaCoO_3_. Phys. B Condens. Matter.

[16] Tanabe Y., Sugano S. (1954). On the absorption spectra of complex ions II. J. Phys. Soc. Jpn..

[17] Ovchinnikov S.G., Orlov Y.S., Nekrasov I.A., Pchelkina Z.V. (2011). Electronic structure, magnetic properties, and mechanism of the insulator–metal transition in LaCoO_3_ taking into account strong electron correlations. J. Exp. Theor. Phys..

[18] Bhide V.G., Rajoria D.S., Reddy Y.S., Rao G.R., Subba Rao G.V., Rao C.N.R. (1972). Localized-to-itinerant electron transitions in rare-earth cobaltates. Phys. Rev. Lett..

[19] Chang C.Y., Lin B.N., Ku H.C., Hsu Y.-Y. (2003). Occurrence and variation of spin-state transitions in La_1-*x*_Eu*_x_*CoO_3_ cobaltates. Chin. J. Phys..

[20] Thornton G., Morrison F.C., Partington S., Tofield B.C., Williams D.E. (1988). The rare earth cobaltates: Localised or collective electron behaviour?. J. Phys. C Solid State Phys..

[21] Yan J.-Q., Zhou J.-S., Goodenough J.B. (2004). Bond-length fluctuations and the spin-state transition in LCoO_3_ (L = La, Pr, and Nd). Phys. Rev. B.

[22] Ivanova N.B., Kazak N.V., Michel C.R., Balaev A.D., Ovchinnikov S.G., Vasil’ev A.D., Bulina N.V., Panchenko E.B. (2007). Effect of strontium and barium doping on the magnetic state and electrical conductivity of GdCoO_3_. Phys. Solid State.

[23] Hoch M.J.R., Nellutla S., van Tol J., Choi E.S., Lu J., Zheng H., Mitchell J.F. (2009). Diamagnetic to paramagnetic transition in LaCoO_3_. Phys. Rev. B.

[24] Yamaguchi S., Okimoto Y., Tokura Y. (1996). Bandwidth dependence of insulator-metal transitions in perovskite cobalt oxides. Phys. Rev. B.

[25] Asai K., Yoneda A., Yokokura O., Tranquada J.M., Shirane G., Kohn K. (1998). Two spin-state transitions in LaCoO_3_. J. Phys. Soc. Jpn..

[26] Ovchinnikov S.G., Orlov Y.S., Dudnikov V.A. (2012). Temperature and field dependent electronic structure and magnetic properties of LaCoO_3_ and GdCoO_3_. J. Magn. Magn. Mater..

[27] Jirák Z., Hejtmanek J., Knižek K., Novak P., Šantava E., Fujishiro H. (2014). Magnetism of perovskite cobaltites with Kramers rare-earth ions. J. Appl. Phys..

[28] Tachibana M., Yoshida T., Kawaji H., Atake T., Takayama-Muromachi E. (2008). Evolution of electronic states in RCoO_3_ (R = rare earth): Heat capacity measurements. Phys. Rev. B.

[29] Dudnikov V.A., Orlov Y.S., Gavrilkin S.Y., Gorev M.V., Vereshchagin S.N., Solovyov L.A., Perov N.S., Ovchinnikov S.G. (2016). Effect of Gd and Sr ordering in A sites of doped Gd_0.2_Sr_0.8_CoO_3−δ_ perovskite on its structural, magnetic, and thermodynamic properties. J. Phys. Chem. C.

[30] Taguchi H. (2002). Electrical properties and spin state of the Co^3+^ ion in (Nd_1−*x*_Gd*_x_*)CoO_3_. Phys. B Condens. Matter.

[31] Yu J., Phelan D., Louca D. (2011). Spin-state transitions in PrCoO_3_ investigated by neutron scattering. Phys. Rev. B.

[32] Umemoto K., Seto Y., Masuda Y. (2005). Structure and magnetic property of Ce*_x_*Eu_1−*x*_CoO_3_ prepared by means of the thermal decomposition of Ce*_x_*Eu_1−*x*_[Co(CN)_6_]·*n*H_2_O. Thermochimicaacta.

[33] Brinks H.W., Fjellvasg H., Kjekshus A., Hauback B.C. (1999). Structure and magnetism of Pr_1− *x*_Sr*_x_*CoO_3−δ_. J. Solid State Chem..

[34] Spinicci R., Faticanti M., Marini P., De Rossi S., Porta P. (2003). Catalytic activity of LaMnO_3_ and LaCoO_3_ perovskites towards VOCs combustion. J. Mol. Catal. A Chem..

[35] Takeda Y., Ueno H., Imanishi N., Yamamoto O., Sammes N., Phillipps M.B. (1996). Gd_1−*x*_Sr*_x_*CoO_3_ for the electrode of solid oxide fuel cells. Solid State Ion..

[36] Liu H., Wu Y.P., Rahm E., Holze R., Wu H.Q. (2004). Cathode materials for lithium ion batteries prepared by sol-gel methods. J. Solid State Electrochem..

[37] Teraoka Y., Zhang H.M., Okamoto K., Yamazoe N. (1988). Mixed ionic-electronic conductivity of La_1−*x*_Sr*_x_*Co_1−*y*_Fe*_y_*O_3−δ_ perovskite-type oxides. Mater. Res. Bull..

[38] Michel C.R., Gago A.S., Guzmán-Colín H., López-Mena E.R., Lardizábal D., Buassi-Monroy O.S. (2004). Electrical properties of the perovskite Y_0.9_Sr_0.1_CoO_3−δ_ prepared by a solution method. Mater. Res. Bull..

[39] Orlov Y.S., Solovyov L.A., Dudnikov V.A., Fedorov A.S., Kuzubov A.A., Kazak N.V., Voronov V.N., Vereshchagin S.N., Shishkina N.N., Perov N.S. (2013). Structural properties and high-temperature spin and electronic transitions in GdCoO_3_: Experiment and theory. Phys. Rev. B.

[40] Solovyov L.A. (2004). Full-profile refinement by derivative difference minimization. J. Appl. Crystallogr..

[41] Kharko O.V., Vasylechko L.O., Ubizskii S.B., Pashuk A., Prots Y. (2014). Structural behavior of continuous solid solution SmCo_1-*x*_Fe*_x_*O_3_. Funct. Mater..

[42] Knížek K., Hejtmánek J., Jirák Z., Tomeš P., Henry P., André G. (2009). Neutron diffraction and heat capacity studies of PrCoO_3_ and NdCoO_3_. Phys. Rev. B.

[43] Orlov Y.S., Dudnikov V.A., Gorev M.V., Vereshchagin S.N., Solov’ev L.A., Ovchinnikov S.G. (2016). Thermal properties of rare earth cobalt oxides and of La_1–*x*_Gd*_x_*CoO_3_ solid solutions. JETP Lett..

[44] Seijas J.G., Prado-Gonjal J., Brande D.A., Terry I., Moran E., Schmidt R. (2016). Microwave-assisted synthesis, microstructure, and magnetic properties of rare-earth cobaltites. Inorg. Chem..

[45] Panfilov A.S., Grechnev G.E., Lyogenkaya A.A., Pashchenko V.A., Zhuravleva I.P., Vasylechko L.O., Hreb V.M., Turchenko V.A., Novoselov D. (2019). Magnetic properties of RCoO_3_ cobaltites (R = La, Pr, Nd, Sm, Eu). Effects of hydrostatic and chemical pressure. Phys. B Condens. Matter.

[46] Vonsovsky S.V. (1971). Magnetism.

[47] Ivanova N.B., Kazak N.V., Michel C.R., Balaev A.D., Ovchinnikov S.G. (2007). Low-temperature magnetic behavior of the rare-earth cobaltites GdCoO_3_ and SmCoO_3_. Phys. Solid State.

[48] Ropka Z., Radwanski R.J. (2003). ^5^D term origin of the excited triplet in LaCoO_3_. Phys. Rev. B.

[49] Zvezdin A.K., Matveev V.M., Mukhin A.A., Popov A.I. (1985). Rare Earth Ions in Magnetically Ordered Crystals.

[50] VanVleck J.H. (1932). The Theory of Electronic and Magnetic Susceptibilities.

[51] Mott N.F., Davis E.A. (1971). Electronic Processes in Non Crystalline Materials.

[52] Berggold K., Kriener M., Zobel C., Reichl A., Reuther M., Müller R., Freimuth A., Lorenz T. (2005). Thermal conductivity, thermopower, and figure of merit of La_1−*x*_Sr*_x_*CoO_3_. Phys. Rev. B.

[53] Scherrer B., Harvey A.S., Tanasescu S., Teodorescu F., Botea A., Conder K., Grundy A.N., Martynczuk J., Gauckler L.J. (2011). Correlation between electrical properties and thermodynamic stability of A CoO_3−δ_ perovskites (A = La, Pr, Nd, Sm, Gd). Phys. Rev. B.

[54] Nagaev E.L., Podel’shchikov A.I. (1996). Phase separation and resistivity jumps in Co compounds and other materials with low-spin-high-spin transitions. J. Phys. Condens. Matter.

[55] Giblin S.R., Terry I., Clark S.J., Prokscha T., Prabhakaran D., Boothroyd A.T., Wu J., Leighton C. (2005). Observation of magnetic excitons in LaCoO_3_. Europhys. Lett..

[56] Vasil’chikova T.N., Kuz’mova T.G., Kamenev A.A., Kaul’ A.R., Vasil’ev A.N. (2013). Spin states of cobalt and the thermodynamics of Sm_1-x_Ca_x_CoO_3-δ_ solid solutions. JETP Lett..

